# Emerging Biologics in Lumbar Disc Degeneration: PRP, Stem Cell Therapy, and Pharmacotherapy in Mobility Restoration and Rehabilitation

**DOI:** 10.26502/fjsrs0097

**Published:** 2026-02-20

**Authors:** Andre Aabedi, Devendra K. Agrawal

**Affiliations:** 1 Department of Translational Research, College of Osteopathic Medicine of the Pacific, Western University of Health Sciences, Pomona, California 91766 USA

**Keywords:** Biologic therapies, Discogenic low back pain, Lumbar disc degeneration (LDD), Mesenchymal stem cells (MSCs), Platelet-rich plasma (PRP), Regenerative medicine, Rehabilitation integration

## Abstract

Lumbar disc degeneration is a leading contributor to chronic low back pain and functional limitation worldwide, driven by progressive extracellular matrix breakdown, disc dehydration, inflammation, and cellular senescence. Conventional treatments—including pharmacotherapy, physical rehabilitation, and surgery—primarily address symptoms rather than the underlying degenerative cascade and often fail to restore disc structure or long-term mobility. Emerging biologic therapies have gained attention for their potential to modify disease progression and promote regeneration. This narrative review examines current evidence surrounding platelet-rich plasma, mesenchymal stem cell therapies, peptide analogs, and evolving pharmacologic agents in the management of lumbar disc degeneration, with particular emphasis on mobility restoration and rehabilitation integration. platelet-rich plasma and mesenchymal stem cell-based interventions demonstrate moderate improvements in pain and functional outcomes with generally favorable safety profiles, though durable structural regeneration remains unproven. Peptide analogs and molecular agents show promising preclinical regenerative and anti-inflammatory effects but lack robust human data and regulatory approval. Pharmacologic strategies targeting inflammatory and catabolic pathways may complement biologic therapies but remain largely investigational. The integration of biologics with structured rehabilitation and progressive loading appears critical for optimizing functional recovery. Despite encouraging advances, significant limitations persist, including heterogeneous protocols, limited high-quality randomized trials, and insufficient long-term data. Future research should prioritize standardization, comparative effectiveness studies, and multimodal treatment models to clarify the role of biologic therapies in restoring mobility and function in lumbar disc degeneration.

## I. Introduction

Degenerative changes in lumbar intervertebral discs represent a primary driver of persistent lower back pain, creating significant worldwide impact through functional impairment, diminished physical capacity, and substantial medical expenditures [1–2]. The disease mechanism centers on disrupted equilibrium between tissue breakdown and rebuilding within the disc, causing deterioration of the extracellular matrix, depletion of proteoglycans and hydration, decreased disc height, and compromised mechanical stability. These degenerative alterations initiate persistent inflammatory responses, abnormal blood vessel formation, and aberrant nerve growth, culminating in heightened pain sensitivity and chronic discomfort [3].

Conventional therapeutic approaches which include rehabilitation exercises, medications and operative procedures such as spinal fusion, disc arthroplasty focus predominantly on symptom management without addressing the fundamental degenerative cascade [4–5]. These interventions fail to regenerate disc architecture or restore physiological function, while surgical alternatives present complications including risks of adverse events, deterioration of neighboring spinal segments, and outcomes that frequently match those achieved through comprehensive non-operative rehabilitation over extended follow-up periods [6–7]. Medication-based treatment demonstrates restricted long-term effectiveness, and minimally invasive techniques (such as epidural steroid administration) yield transient benefit only [8].

The scientific basis for biologic interventions in mobility enhancement rests on their capacity to alter disease trajectory and stimulate disc tissue regeneration. Biologic therapies including platelet-derived growth factor concentrates in platelet-rich plasma (PRP), multipotent stromal cells (MSCs), and bioactive peptide compounds seek to reconstitute extracellular matrix (ECM) organization, dampen inflammatory processes, and facilitate tissue healing [9]. Preliminary human trials reveal enhanced pain relief, functional restoration, and improved well-being, with PRP and MSCs exhibiting regenerative properties and cartilage-like tissue formation within affected discs [10–12]. These treatment modalities may target fundamental etiologic factors of degeneration, presenting opportunities for genuine mobility restoration and biomechanical recovery beyond simple symptomatic control. [13–14].

Nevertheless, uncertainties persist concerning durability of treatment effects, safety profiles, and appropriate candidate identification, requiring additional investigation before broader clinical implementation [15–16]. In conclusion, biologic therapeutics constitute an encouraging therapeutic evolution in managing lumbar disc degeneration, addressing core pathophysiology with potential to restore both mobility and functional capacity.

## II. Key Pathobiology Relevant to Regeneration

Disc dehydration, inflammation, and matrix breakdown are central pathological processes in lumbar disc degeneration (LDD), each critically influencing the potential for regeneration and the efficacy of emerging biologic therapies. Disc dehydration is an early hallmark of degeneration, resulting from the loss of proteoglycans in the nucleus pulposus, which impairs the disc’s ability to retain water and maintain height and biomechanical function. This dehydration leads to reduced disc elasticity and increased susceptibility to mechanical stress, further accelerating degeneration and limiting the microenvironment’s capacity for regeneration [5][9][17].

**Figure 1:**
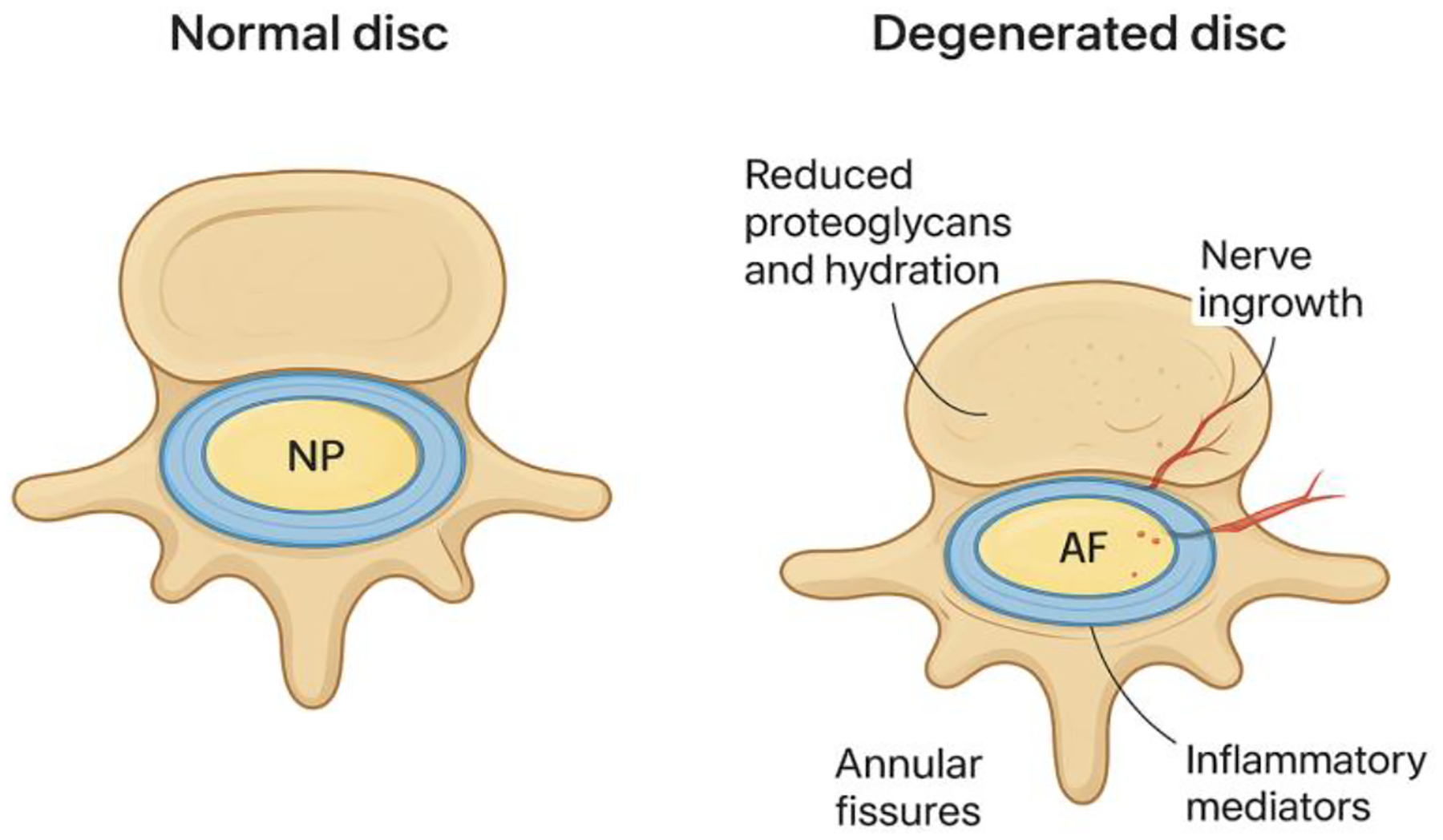
Schematic comparison of a healthy versus degenerated lumbar intervertebral disc demonstrating loss of proteoglycan content and hydration, annular fissuring, inflammatory mediator release, and aberrant nerve ingrowth associated with disc degeneration. AF, annulus fibrosus; NP, nucleus pulposus.

Inflammation plays a dual role in disc pathology. Chronic inflammation, driven by immune cell infiltration (macrophages, T cells, B cells) and pro-inflammatory cytokines (IL-1β, TNF-α, IL-6), activates signaling pathways such as NF-κB and MAPK, promoting disc cell apoptosis and extracellular matrix (ECM) degradation (Figure 1). This inflammatory milieu not only exacerbates tissue breakdown but also impairs the survival and function of endogenous and transplanted regenerative cells. However, targeted modulation of inflammation—using anti-inflammatory drugs, biologics, or gene editing—may create a more favorable environment for disc repair [18].

Matrix breakdown involves the loss of key ECM components, including collagen and aggrecan, due to increased activity of matrix metalloproteinases and aggrecanases. This disrupts disc structure, reduces mechanical integrity, and impairs cell-matrix interactions necessary for regeneration. Cellular senescence and programmed cell death further diminish the disc’s reparative capacity [17].

**Figure 2:**
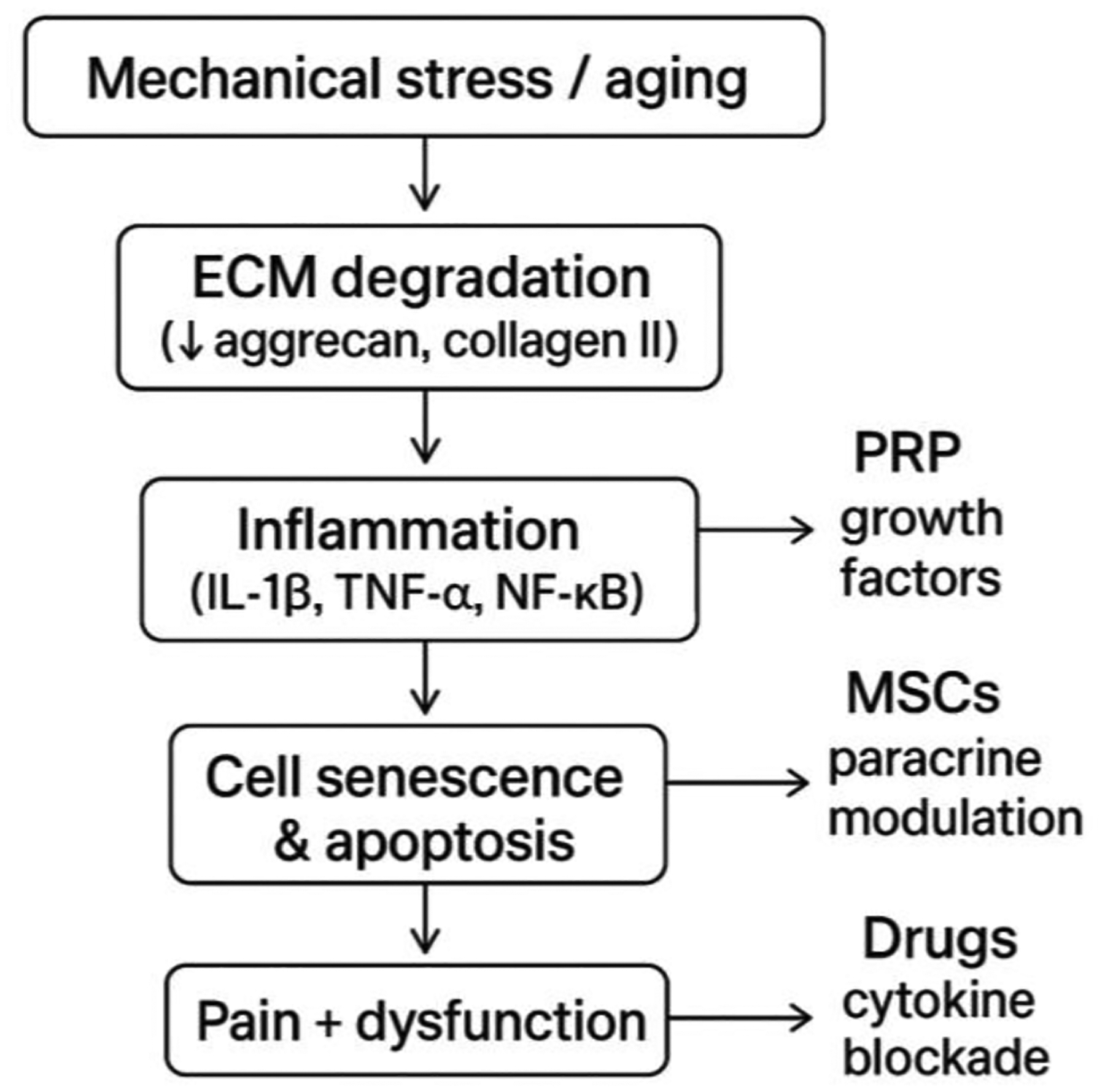
Conceptual flow diagram illustrating the pathobiologic cascade of lumbar disc degeneration from mechanical stress and aging to extracellular matrix degradation, inflammation, cellular senescence, and pain, with key therapeutic intervention points for PRP, mesenchymal stem cells, and pharmacologic agents. ECM, extracellular matrix; PRP, platelet-rich plasma.

In the context of regeneration, these pathological changes present both challenges and therapeutic targets. Biologics such as platelet-rich plasma (PRP) and stem cell therapies aim to restore disc hydration, suppress inflammation, and promote matrix synthesis. PRP delivers growth factors that stimulate cell proliferation and ECM regeneration, while mesenchymal stem cells and their exosomes modulate inflammation and support tissue repair (Figure 2) [19]. Peptide analogs and pharmacotherapies targeting inflammatory and catabolic pathways are under investigation to further enhance the regenerative microenvironment [20].

## III. Platelet-Rich Plasma (PRP)

Platelet-rich plasma therapy for lumbar disc degeneration works through its bioactive constituents, particularly growth factors including platelet-derived growth factor (PDGF), transforming growth factor-beta (TGF-β), and vascular endothelial growth factor (VEGF), which facilitate cellular proliferation, extracellular matrix restoration, and tissue repair. Additionally, PRP contains anti-inflammatory mediators that may help regulate the inflammatory environment, potentially alleviating pain and slowing degenerative changes in the intervertebral disc [12], [21]. Laboratory investigations have shown that PRP can enhance disc cell function and matrix production, while patient data indicate pain reduction and functional improvements in individuals with discogenic lower back pain [22], [23].

The strongest clinical support exists for intradiscal PRP delivery in treating discogenic pain, with multiple prospective investigations and randomized controlled trials demonstrating meaningful and durable improvements in pain and physical function extending up to 5–9 years [24–25]. Systematic reviews and meta-analyses show that intradiscal PRP injections achieve at least 50% pain reduction in roughly half of patients at the 6-month mark, alongside notable functional gains [23]. By comparison, perispinal applications (such as facet joint or epidural injections) have received less research attention for discogenic pain, and existing evidence suggests intradiscal administration may more effectively address disc pathology itself [12]. Nevertheless, head-to-head studies comparing intradiscal versus perispinal techniques remain sparse, and additional investigation is warranted to determine the most effective delivery method [21].

A significant challenge in PRP therapy lies in the marked heterogeneity of preparation methods, platelet concentrations, and injection protocols, resulting in variable clinical results and complicating comparisons across studies [3]. This absence of standardized PRP preparation and documentation impedes reproducibility and the development of evidence-based guidelines. Furthermore, the strength of available evidence remains modest, as most studies are limited in size, heterogeneous in design, and lack rigorous control groups [26]. Complications occur rarely but may include infection and temporary worsening of pain [11]. Moving forward, research priorities should emphasize protocol standardization, exploration of dose-response relationships, and implementation of well-designed randomized trials to more clearly establish PRP’s utility in enhancing mobility and supporting rehabilitation for lumbar disc degeneration [22].

## IV. Stem Cell Therapy

Stem cell therapy, especially utilizing MSCs, represents a prominent biologic strategy being explored for lumbar disc degeneration, with preliminary clinical trials demonstrating encouraging yet inconclusive findings. MSCs are typically obtained from bone marrow, adipose tissue, and more recently, umbilical cord tissue, each offering unique benefits concerning harvesting ease, differentiation capabilities, immune compatibility, and ethical implications. For instance, umbilical cord-derived MSCs possess robust proliferative potential and minimal immunogenicity, rendering them suitable for allogeneic use, though their viability in the harsh disc microenvironment poses ongoing difficulties [27].

At the mechanistic level, MSCs facilitate disc regeneration through two principal mechanisms: direct transformation into disc-type cells (nucleus pulposus and annulus fibrosus cells) and paracrine activity. The paracrine mechanism—driven by released factors and exosomes—regulates inflammatory processes, enhances endogenous cell proliferation, prevents programmed cell death, and aids extracellular matrix reconstruction, which is now understood as the predominant therapeutic mechanism in living systems [19], [28]. These processes can result in pain alleviation and enhanced function, as evidenced in multiple preliminary clinical investigations [23].

Clinical data from early-stage trials and meta-analytic reviews suggest that intradiscal MSC administration is typically safe and may yield meaningful reductions in pain and disability measures for individuals with discogenic lower back pain. Both autologous and allogeneic MSC approaches have demonstrated feasibility and safety profiles, with certain studies documenting persistent benefits across multiple years and minimal complication rates [29]. Nevertheless, the overall evidence quality remains limited, as some trials have failed to demonstrate advantages over placebo or saline controls, underscoring the necessity for larger, rigorously controlled investigations [3].

Primary obstacles to clinical implementation include substantial costs, cell survival within the avascular and inhospitable disc environment, and intricate regulatory barriers. Cell viability is compromised by inadequate nutrient availability and reduced oxygen levels in degenerated discs, driving research into biomaterial scaffolds and tissue engineering approaches to improve MSC retention and therapeutic effectiveness [30]. Regulatory challenges focus on standardizing cell processing methods, ensuring patient safety, and establishing consistent therapeutic outcomes, which currently limit broader clinical utilization [31]. Expenses remain considerable due to requirements for cell isolation, expansion, and administration, necessitating additional research to refine protocols and decrease overall costs.

## V. Peptide Analogs and Molecular Agents

Peptide analogs including BPC-157, thymosin beta-4, and GHK-Cu represent novel molecular candidates showing potential regenerative properties for musculoskeletal and intervertebral disc degeneration, though existing evidence remains predominantly preclinical, with substantial safety and regulatory constraints persisting.

BPC-157 is a stabilized gastric pentadecapeptide exhibiting diverse biological effects in rodent models of tissue damage, encompassing tendon, ligament, muscle, and bone repair. Preclinical investigations reliably demonstrate enhanced healing rates, angiogenic modulation, and cellular protective actions, with minimal documented adverse effects and favorable safety profiles in animal research [32]. The therapeutic mechanisms of BPC-157 involve nitric oxide pathway regulation and anti-inflammatory activity, which may hold relevance for restoring the disc microenvironment [33–34]. Nevertheless, human clinical data remain absent, and BPC-157 lacks FDA approval or authorization from other major regulatory authorities for medical application due to inadequate clinical trial evidence. Its temporary inclusion on World Anti-doping Agency’s monitoring list underscores persistent safety and ethical considerations, particularly concerning unsupervised administration and uncontrolled online distribution [32].

Thymosin beta-4 and GHK-Cu are similarly being examined for their regenerative and anti-inflammatory characteristics. Thymosin beta-4 facilitates cell migration, angiogenesis, and tissue restoration, whereas GHK-Cu functions as a copper-binding peptide involved in wound healing and inflammatory modulation. While both have demonstrated potential in soft tissue and dermal repair models, specific evidence addressing lumbar disc degeneration remains limited, and neither compound possesses substantial clinical trial support in this application. Their mechanisms—including anti-apoptotic and anabolic activities—correspond with molecular targets implicated in disc degeneration, yet clinical implementation remains at an early developmental stage [35].

Constraints and safety/regulatory considerations encompass the absence of large-scale, controlled human investigations, uncertain long-term safety characteristics, and ambiguous regulatory standing. None of these peptides currently hold approval for lumbar disc degeneration treatment, and their application remains experimental. The lack of standardized dosing protocols, administration techniques, and extended monitoring further hinders clinical translation. Regulatory authorities mandate rigorous demonstration of efficacy and safety before authorization, and available data have not yet satisfied these requirement [34], [36].

## VI. Pharmacotherapy

Treatment with medications for lumbar disc degeneration is currently focused on relieving symptoms, though novel therapies aimed at modifying disease progression—such as anti-cytokine and anti-fibrotic compounds—are being studied and may work effectively when combined with biological treatments like platelet-rich plasma and stem cell approaches.

Existing symptom-focused medications consist mainly of non-steroidal anti-inflammatory drugs (NSAIDs), acetaminophen, muscle relaxants, and selectively prescribed opioids or gabapentinoids. While these drugs help manage pain and inflammation, they do not change the degenerative process itself [37]. Corticosteroid injections provide temporary relief but offer limited sustained benefits [13].

New disease-modifying pharmaceutical approaches focus on the molecular mechanisms driving disc degeneration. Persistent inflammation driven by cytokines like IL-1β, TNF-α, and IL-6 plays a crucial role in the initiation and pathogenesis of lumbar disc degeneration. Experimental anti-cytokine treatments—including agents that block these cytokines and their signaling cascades (such as NF-κB and MAPK pathways)—are being studied for their potential to dampen inflammation and decelerate degenerative changes [35]. Compounds that combat fibrosis and senolytic drugs, which eliminate senescent cells and address fibrotic changes in the disc, are currently in preclinical and early-phase clinical testing [38]. Additional molecular targets being investigated include MMPs, ADAMTS enzymes, and neurotrophic factors that contribute to pain amplification and extracellular matrix breakdown [10].

The combination of pharmacological and biological interventions represents a promising research direction. Biological agents like PRP and mesenchymal stem cells offer anti-inflammatory and tissue-regenerating properties while potentially amplifying the effectiveness of drug therapies by altering the disc’s cellular environment [4], [24]. As an example, pairing anti-cytokine medications with cellular therapies could simultaneously reduce inflammation and stimulate matrix restoration and cell viability. Preliminary clinical data indicate that biological treatments can decrease pain and enhance physical function, and their pairing with targeted drug therapy may yield improved clinical results [9].

## VII. Integration with Rehabilitation

Biological therapies including platelet-rich plasma, stem cell treatments, and peptide analogs are being progressively incorporated into rehabilitation programs for lumbar disc degeneration, with the goal of promoting tissue repair, alleviating pain, and restoring function. These interventions work best not in isolation but when paired with systematic physical therapy and progressive loading strategies that create favorable biomechanical conditions for disc recovery and functional improvement [10–11]. Following biologic injection, patients are typically advised to begin early mobilization and physical therapy to maintain conditioning and support disc nutrition through movement. Progressive loading in terms of the stepwise advancement of activity demands works in concert with the reparative effects of biologics by encouraging tissue adaptation and neuromuscular refinement. Physical therapy emphasizes core strengthening, mobility enhancement, and systematic progression toward normal activities, complementing the anti-inflammatory and regenerative capabilities of biological agents [3],[12]. For instance, PRP and stem cell interventions may diminish pain and inflammation, enabling patients to engage more effectively in rehabilitation and attain superior functional gains.

Most treatment protocols suggest a short period of modified activity (generally 24–72 hours) following injection, after which low-intensity exercises and physical therapy are gradually introduced within the first week. More demanding or high-impact activities are typically postponed for 2–4 weeks, with advancement determined by pain levels, functional status, and clinical judgment [10]. Clinical evidence demonstrates lasting improvements in pain and function after biologic therapies, with benefits persisting up to 5–9 years in certain PRP-treated groups [3]. Measures of functional capacity, disability indices, and patient satisfaction show consistent enhancement when biologics are combined with rehabilitation compared to either approach used independently [12]. Nevertheless, the evidence base remains limited to moderate in quality, highlighting the need for additional rigorous studies to establish optimal treatment protocols and confirm long-term effectiveness [24].

## VIII. Comparative Analysis and Future Directions

Current evidence indicates that biological treatments for lumbar disc degeneration including platelet rich plasma, stem cell therapy, peptide analogs, and novel pharmacological approaches offer potential for pain reduction and functional enhancement, though their relative effectiveness, safety profiles, and clinical feasibility remain inadequately characterized with significant knowledge gaps. PRP and stem cell therapies represent the most extensively investigated modalities, with PRP showing durable pain and functional improvements in multiple prospective studies and randomized trials, including benefits lasting 5–9 years in some patients [23]. Success rates for PRP and mesenchymal stem cells are comparable, with roughly 50–55% of patients experiencing meaningful pain relief at six months, though evidence quality is limited and outcomes vary considerably [39]. Both approaches demonstrate acceptable safety with infrequent serious complications, although isolated cases of infection have occurred [11]. Clinical implementation is constrained by requirements for specialized expertise, equipment, and substantial costs that limit accessibility and patient uptake, particularly for stem cell interventions. Peptide analogs and biomaterial based strategies such as self-assembling peptide hydrogels and biomimetic compounds remain in earlier developmental phases, with preclinical research indicating capacity for disc regeneration, stem cell preservation, and improved disc structure and mechanics, though clinical application awaits validation and long term safety assessment [40–41]. Pharmacotherapy continues to address symptoms rather than underlying degeneration, with experimental approaches like senolytics and ferroptosis inhibitors currently lacking substantial clinical evidence [17].

Comparative evaluation reveals that while PRP and MSCs demonstrate similar short-term results, neither achieves complete disc structural restoration, and long-term trajectories remain unclear [42]. Peptide analogs and biomaterials may augment regenerative outcomes when combined with cellular therapies, but direct comparative studies are absent [41]. Safety profiles appear generally acceptable, though rare adverse events and uncertain long-term risks necessitate ongoing vigilance. Critical evidence deficiencies include insufficient high-quality, adequately powered randomized controlled trials, sparse long-term safety and effectiveness data, and minimal direct comparisons between treatment modalities. Standardization of biological product preparation, administration techniques, and outcome assessment is essential [29]. Future research priorities should emphasize methodologically rigorous trials with well-defined eligibility criteria, extended follow-up periods, and comparative effectiveness analyses. Advancing the field and establishing biological therapies as credible options for mobility restoration and rehabilitation in lumbar disc degeneration will require integration of molecular discoveries, biomaterial advances, and patient-centered outcome measures [43].

## IX. Conclusion

Biologic therapies represent a promising shift in the management of lumbar disc degeneration by targeting the underlying biological mechanisms—such as inflammation, matrix breakdown, and cellular senescence—that traditional treatments fail to address. Platelet-rich plasma and mesenchymal stem cell therapies have the strongest evidence to date, showing moderate improvements in pain and function with generally favorable safety profiles, though long-term structural regeneration remains unproven. Peptide analogs, biomaterial scaffolds, and emerging molecular agents offer additional regenerative potential but remain predominantly in early research stages. Rehabilitation plays a critical synergistic role, as biologics appear most effective when combined with structured physical therapy and progressive loading protocols that support tissue healing and mobility restoration.

Despite encouraging progress, significant gaps remain, including variable preparation methods, limited high-quality randomized trials, and inadequate long-term data. Standardization of biologic products and delivery techniques, along with rigorous comparative studies, will be essential to establishing reliable clinical guidelines. With continued scientific refinement and validation, biologic therapies may ultimately provide durable improvements in function and quality of life by addressing the root causes of disc degeneration rather than offering temporary symptom relief.

## Key points

- Lumbar disc degeneration is characterized by disc dehydration, inflammation, extracellular matrix degradation, and cellular senescence, leading to chronic pain and impaired mobility.
- Conventional treatments primarily provide symptomatic relief and do not reverse or halt the underlying degenerative process.
- Platelet-rich plasma has the strongest clinical evidence among biologics, demonstrating moderate and sometimes durable improvements in pain and function.
- Mesenchymal stem cell therapies show potential regenerative and immunomodulatory effects but face challenges related to cost, cell survival, and regulatory complexity.
- Peptide analogs and molecular agents exhibit promising preclinical regenerative properties but lack sufficient clinical validation for routine use.
- Pharmacologic therapies remain largely symptom-focused, although disease-modifying targets such as cytokine inhibition and senolytics are under investigation.
- Biologic therapies appear most effective when integrated with structured rehabilitation and progressive mechanical loading strategies.
- Current evidence is limited by heterogeneity in biologic preparation, delivery techniques, and outcome measures.
- High-quality randomized trials, long-term follow-up, and standardized protocols are essential to establish biologic therapies as reliable options for mobility restoration in lumbar disc degeneration.
